# Supplementary material for: A polycistronic system for multiplexed and precalibrated expression of multigene pathways in fungi
Source: Nat Commun. 2023 Jul 17;14:4267. doi: 10.1038/s41467-023-40027-0 (PMC10352335; doi:10.1038/s41467-023-40027-0)
Supplement: Supplementary file 1 — Supplementary Information [file 41467_2023_40027_MOESM1_ESM.pdf]

A polycistronic system for multiplexed and precalibrated expression of multigene pathways in fungi

Yue *et al.*

Supplementary Note 1. Selection of driver genes for the HACKing system and considerations for the design of HACKing experiments

Proteomics analysis to determine the abundance of Saccharomyces cerevisiae proteins

A total of 3,807 proteins were detected by quantitative proteome analysis in exponential growth phase *S. cerevisiae* CEN.PK2-1D cells, grown in YPD medium at 30 °C with shaking at 250 rpm for 24 h (Methods). The relative protein abundances of these proteins showed large differences (Supplementary Data 1). We selected 286 proteins whose intensity-based absolute quantification (iBAQ) yielded abundancy values higher than 5×10^5 (Supplementary Data 1, yellow highlight). Some of the encoding genes for these proteins, such as *TDH3*, *CCW12*, and *TEF1* are known to be controlled by strong promoters that are widely used in synthetic biology [1]. These 286 genes were investigated for CRISPR/Cas9-mediated integration sites for the construction of *IGG6*-mediated bicistronic cassettes.

gRNA screening

Generally, an appropriate gRNA sequence for a protospacer preceding a PAM sequence, and homologous targeting arms of a minimum of 30 bp are necessary for CRISPR/Cas9-mediated integration of donor DNA in *S. cerevisiae* [2,3]. We planned to amplify the donor DNA by a simple one-step PCR, where integration (knock-in) of the donor DNA would lead to the *IGG6*-mediated coupling of the gene of interest (GOI) to the driver gene. For this, the CRISPR/Cas9-mediated cleavage site should be close to the stop codon of the endogenous driver gene. Thus, potential gRNAs were identified for 104 of the 286 genes selected in the quantitative proteomics experiment, with a CRISPR/Cas9 cleavage site that is less than 5 bp away from the stop codon of the driver gene (Supplementary Data 2).

For all gene integrations designed as shown in Fig. 3, the PAM region is located close to the stop codon and often within the ORF of the endogenous driver gene. Thus, preserving the

sequence of the driver gene means that the PAM sequence is often not eliminated during our engineering. However, the length between the cleavage site and the stop codon was designed to be at most 5 base pairs (Supplementary Data 2), which means that after the donor DNA is integrated, at most 8 base pairs would be left from the protospacer. Considering that a contiguous stretch of at least 13 base pairs is required for annealing between the gRNA and the target DNA site proximal to the PAM for efficient target cleavage [4], the remaining PAM-protospacer sequence is too short to be functional.

Donor DNA design

All the donor DNA fragments were obtained by one-step PCR using primers of 59-90 nucleotides in length. The primers contained the complementary regions to amplify the coding sequence of the GOI; any signal or targeting sequences (if desired) fused to the GOI; the appropriate *IGG* sequence (in most cases, *IGG6*) designed between the stop codon of the driver gene and the start codon of the GOI; and the targeting homologous arms (TAs) for integration.

Application of HACKing for expression of multiple genes encoding a biosynthetic pathway

The expression levels of GOI integrated as *IGG6*-coupled bicistrons show a positive correlation with those of their endogenous driver gene partners (Supplementary Fig. 10). Thus, expression of GOI in multistep pathways can be individually precalibrated by linking them to driver genes of the host organism that show appropriate protein abundances under the desired culture conditions. These can include drivers with the highest expression level, such as *TDH3* or *TEF1* in our experiments. In other cases, the expression of some GOI can be moderated by hitching them to drivers with lower expression, such as *GPD1* or *SPE3*. Similarly, users of the HACKing system may choose drivers with specific expression patterns, such as those that are predominantly translated in the early or conversely, the late growth phases; or those that respond to metabolic stimuli such as the presence of inducers, other metabolites or media

components, different temperature regimes, pH changes, and so on. It is important to consider the fermentation process parameters that are planned to be utilized for the production of the target metabolite of the incoming biosynthetic pathway, and conduct the proteomics analysis under those conditions to identify driver genes showing the desired translation pattern.

Supplementary Method 1. Construction of expression cassettes for the optimization and evaluation of *IGG* sequences

To evaluate the applicability of *IGG1* in *S. cerevisiae*, a FLAG tag was appended to the *C*-terminus of TDH3 first. DNA fragments of *TDH3*, *TRP1*, and T_{ADH2} were inserted into pJET1.2 to produce the cloning vector pJET1.2-TDH3-FLAG (Supplementary Data 7 for plasmids used in this study). Briefly, 0.6-kb upstream and downstream targeting arms for the *TDH3* gene were amplified from the genomic DNA of *S. cerevisiae* CEN.PK2-1D using primer sets TDH3-uF1 + TDH3-FLAG-R and TDH3-dF1 + TDH3-dR1, respectively (Supplementary Data 9 for primer sequences). *TRP1* and T_{ADH2} were amplified from YEpADH2p-TRP as the template using primer sets Trp1-F1 + Trp1-R4 and Tadh2-F1 + Tadh2-R1, respectively [5]. All amplicons were combined and ligated into pJET1.2 by In-Fusion cloning technology (Vazyme, China) to produce pJET1.2-TDH3-FLAG. Primer set TDH3-uF2 + TDH3-dR2 was used to amplify the DNA fragment TDH3-FLAG-T_{ADH2}-TRP1-dTDH3. Then, primer sets TDH3-uF1 + FLAG-R1 and Tadh2-F1 + TDH3-dR1 were used to amplify TDH3-FLAG-IGG1 and T_{ADH2}-TRP1-dTDH3 from pJET1.2-TDH3-FLAG as the template. The primer set GFP-F1 + GFP-Tadh2-R1 was used to amplify a 0.7-kb DNA segment of the *GFP* gene from pEASY-Blunt-GFP [6]. All amplicons were combined and ligated into pJET1.2 by In-Fusion cloning technology (Vazyme, China) to produce pJET1.2-TDH3-FLAG::IGG1-GFP. Primer set TDH3-uF2 + TDH3-dR2 was used to amplify the GFP expression cassette TDH3-IGG1-GFP.

To optimize the sequence of IGG, primer sets TDH3-uF1 + FLAG-R2 and GFP-F2 + TDH3-dR1 (Supplementary Data 9) were used with template pJET1.2-TDH3-FLAG::IGG1-GFP (Supplementary Data 7) to amplify TDH3-FLAG-IGG2 and IGG2-GFP-T_{ADH2}-TRP1-dTDH3, respectively. The two amplicons were combined and ligated with pJET1.2 by In-Fusion cloning technology (Vazyme, China) to produce pJET1.2-TDH3-FLAG::IGG2-GFP. Primer set TDH3-uF2 + TDH3-dR2 was used to amplify the GFP expression cassette TDH3-IGG2-GFP. Similar procedures were used with the appropriate primers to obtain the GFP expression cassettes TDH3-IGG3-GFP to TDH3-IGG11-GFP, and TDH3-GFP, TDH3-fusion-GFP as the controls. To construct ΔTDH3::P_{TDH3}-GFP as a control, primer sets TDH3-uF3 + TDH3-uR3 and GFP-TDH3-F1 + TDH3-dR1 were used to amplify the upstream region of TDH3 and GFP-T_{ADH2}-TRP1-dTDH3, respectively. The two amplicons were combined and ligated with pJET1.2 by In-Fusion cloning technology (Vazyme, China) to produce pJET1.2-uTDH3-GFP. Primer set TDH3-uF4 + TDH3-dR2 was used to amplify DNA fragment uTDH3-GFP-T_{ADH2}-TRP1-dTDH3.

To determine the translation pattern of the synthetic bicistron constructed with *IGG6*, *GFP* and *mCherry*, these sequences were cloned into pRS425 to construct expression vector pRS425-P_{TEF1}-mCherry::IGG6-GFP, pRS425-P_{TEF1}-mCherry-fusion-GFP and pRS425-P_{TEF1}-mCherry::GFP (Supplementary Data 7). Primer sets Mcherry-FLAG-pRS425-F + Mcherry-MDH3-GAP-R and GFP-SV40-GAP-MDH3-F + GFP-SV40-R (Supplementary Data 9) were used to amplify m*Cherry* with the *IGG6* and MDH3 peroxisome targeting sequence and *GFP* with *MDH3*, *IGG6* and SV40 nuclear signal sequence, respectively. Primer set GFP-MDH3-mcherry-F + GFP-end-R was used to amplify GFP containing linker sequence with *mCherry* (no stop codon); primer set GFP-MDH3taa-mcherry-F + GFP-end-R was used to amplify *GFP* linked with *mCherry* containing stop codon; primer set CYC1t-GFP-F + CYC1t-p-R was used to amplify the terminator. Primer set pRS425-SV40-F + pRS425-TEF1p-FLAG-R was used to amplify the pRS425 plasmid. All amplicons were

combined and ligated by In-Fusion cloning technology (Vazyme, China) to produce pRS425-P_{TEF1}-mCherry::IGG6-GFP, pRS425-P_{TEF1}-mCherry-fusion-GFP and pRS425-P_{TEF1}-mCherry::GFP. Further, a translation blocking sequence (TBS) was introduced upstream of the start codon of *TDH3*; upstream of the start codon of *GFP*; or downstream of the stop codon of *TDH3*, respectively. The TBS DNA fragments, with or without overlaps to *IGG6* or the upstream sequences of *TDH3*, were amplified from custom synthetized TBS with primer sets TBS-R + 5#-TBS-F1 (or TBS-F or TBS-TDH3-F), respectively [7]. Primer sets TDH3-uF1 + FLAG-TBS-R or FLAG-R6 and TDH3-dR1 + TBS-5#-GFP-F1 or GFP-TBS-F1 were used with pJET1.2-TDH3-FLAG::IGG6-GFP as the template to amplify DNA fragments TDH3-FLAG, IGG6-GFP-T_{ADH2}-TRP1-dTDH3 and GFP-T_{ADH2}-TRP1-dTDH3, respectively. Primer set TDH3-TBS-F1 + TDH3-dR1 was used with chromosomal DNA from yeast transformed with TDH3-FLAG::IGG6-GFP as the substrate, to amplify a 3.6-kb DNA fragment of *TDH3* (from start codon)-FLAG-IGG6-GFP-T_{ADH2}-TRP1-dTDH3. DNA fragments with overlaps were combined and ligated with pJET1.2 by In-Fusion cloning technology (Vazyme, China). Primer set TDH3-uF2 + TDH3-dR2 was used to amplify the *GFP* expression cassettes of TDH3-FLAG::TBS-IGG6-GFP and TDH3-FLAG::IGG6-TBS-GFP. Primer set TDH3-uF4 + TDH3-dR2 was used to amplify the *GFP* expression cassette TBS-TDH3-FLAG::IGG6-GFP.

Supplementary Method 2. Preparation of plasmids for the construction of *Pichia pastoris*, *Yarrowia lipolytica*, and *Aspergillus nidulans* GFP-expressing strains

To test the functionality of *IGG6* in *P. pastoris*, *Y. lipolytica*, and *A. nidulans*, homologous recombination was adopted to integrate the *IGG6-GFP* cassette with a selection marker into the genome of these fungi. Primer sets Pp TDH3-up-F + Pp TDH3-up-R and Pp TDH3-down-F + Pp TDH3-down-R were used with template *P. pastoris* GS115 genomic DNA to amplify the upstream and downstream homologous arms of driver *PpTDH3*, respectively. Primer sets YL TEF1-up-F + YL TEF1-up-R and YL TEF1-down-F + YL

TEF1-down-R were used with template *Y. lipolytica PO1f* genomic DNA to amplify the upstream and downstream homologous arms of driver *YlTEF1*, respectively. Primer sets BleoR-F + BleoR-R and Yl His4-F + Yl His4-R were used to amplify marker genes for *Pichia pastoris* and *Yarrowia lipolytica*, respectively. Primer sets yeGFP-IGG6-F1 + yeGFP-R1 and TtrpC-yeGFP-F1 + TtrpC-R1 were used to amplify *IGG6-GFP* and *TtrpC*, respectively. Primer sets pHR-F + pHR-R were used with psgRNA as the template to amplify the pHR plasmid. Primer sets pyrG-TtrpC-F1 + pyrG-R1 and Ori-F1 + ampR-R1 were used with pYH-WA-pyrG [8] as the template to amplify *pyrG* and the pYH plasmid, respectively. Primer sets NpgA-uF1 + NpgA-uR1 and NpgA-pyrG-dF1 + NpgA-ori-dR1 were used with chromosomal DNA from *A. nidulans* A1145 ΔEMΔST as the substrate, to amplify the upstream and downstream targeting arms for the *NpgA* gene, respectively. DNA fragments with overlaps were combined and ligated together by In-Fusion cloning technology (Vazyme, China) to produce pHR-PpTDH3-IGG6-GFP, pHR-YlTEF1-IGG6-GFP, and pYH-NpgA-IGG6-GFP-pyrG as donor plasmids.

Supplementary Method 3. Preparation of plasmids and donor DNAs for the construction of squalene and mogrol cell factories using the HACKing system

To construct pCas9-based plasmids for GTR-CRISPR integration [3], DNA fragments of gRNAs and *ScUra* were inserted into pCas9 to produce the cloning vectors pCas-SEI1, pCas-SEI2, and pCas-MEI1~MEI5 (Supplementary Data 7 for plasmids used in this study). Briefly, 0.2-kb gRNA-expressing cassettes were amplified from the plasmid psgtRNA using primer sets sgRNA-SEI/MEI n-n-BsaI-F + sgRNA-SEI/MEI n-n-BsaI-R; and 1.5-kb ScUra-expressing cassettes were amplified from the plasmid pScURA3 using primer sets ScUra- SEI/MEI n-n-BsaI-F + ScUra-BsaI-R, respectively (Supplementary Data 9 for primer sequences). The amplicons were combined as designed and ligated into pCas by Golden Gate assembly (New England Biolabs, USA) to produce plasmids pCas-SEI1, pCas-SEI2, pCas-MEI1, pCas-MEI2, pCas-MEI3, pCas-MEI4, and pCas-MEI5.

The donor DNA containing TAs, *IGG6* and GOIs for genomic integration were obtained by one-step PCR. With the primer sets driver-GOI-IGG6-F + driver-GOI-MDH3-R, DNA fragments of donor DNA for *ERG12*, *ERG8*, *ERG19*, *ERG20*, *ERG9*, *IDI1* were amplified from genomic DNA of *S. cerevisiae* CEN.PK2-1D; while DNA fragments of donor DNA for *EfmvaE*, *EfmvaS*, *AtCPR*, *SgCYP87D18*, *SgCDS*, *SgSQE1*, *SgEPH3* were amplified from pRS414-based plasmids, respectively.

Supplementary Table 1. The DNA sequences of IRESs and 2A peptides used in this study.

Name	DNA sequence (5’->3’)
IRES-8	AAGGGCTGCAAATATCTG
IRES-10	GGCCTGGGCGCGGCTGAG
IRES-32	GGGCAGCATCGCTGCGAC
IRES-40	AGGGGGGCGGGGAGTGAT
IRES-41	GATGCTGGGGTTTTGCAT
IRES-47	AGGAGAGGCTGGTTGCTA
ERBV-1 2A	GGTTCTGGTGGTGCTACCAATTTTTCTTTGTTGAAATTGGCTGGTGATGTTGAATTGAATCCAGGTCCA
P2A	GGTTCCGGTGCCACTAACTTCTCTTTGTTGAAGCAAGCTGGTGACGTTGAAGAAAACCCAGGTCCA

Supplementary Table 2. Bicistronic expression units for squalene production.

Incoming pathway gene	Endogenous driver gene, strain HCS1
EfmvaE	*TDH2*
EfmvaS	*TIF1*
ERG12	*RPS10A*
ERG8	*CYS3*
ERG19	*RPS25A*
IDI1	*PFY1*
ERG20	*RPS0A*
ERG9	*RPL38*

Supplementary Table 3. Bicistronic expression units for mogrol production.

Incoming pathway gene	Endogenous driver gene		
	**Strain HCM1**	**Strain HCM2**	**Strain HCM3**
SgSQE1	*TDH1*	*TRR1*	*THI20*
SgCDS	*TEF1*	*HSP104*	*TMA17*
SgEPH3	*ILV5*	*QCR2*	*HBN1*
SgCYP87D18	*TDH3*	*TDH3*	*TDH3*
AtCPR	*SOD1*	*SOD1*	*SOD1*

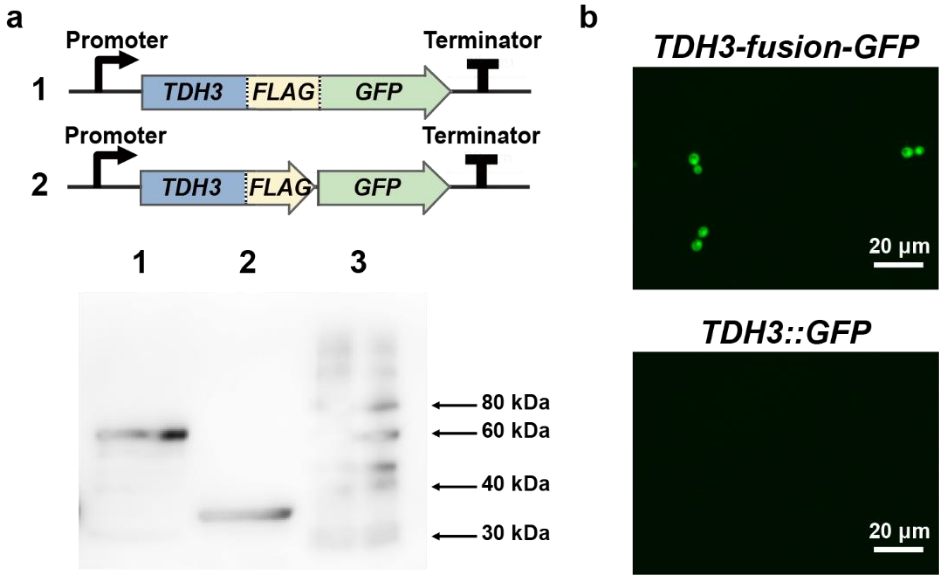

Supplementary Fig. 1. Expression of Tdh3p and GFP in the *TDH3-fusion-GFP* and *TDH3::GFP* transformants.

TDH3-fusion-GFP was constructed by replacing the stop codon and 45 bp of the 3' untranslated region (UTR) of the *TDH3* gene with a DNA fragment for *FLAG(no stop codon)-GFP-*T_{ADH2}*-TRP1* by gene knock-in via homologous recombination, creating a gene fusion that encodes Tdh3p-FLAG-GFP (including an internal FLAG tag). *TDH3::GFP* was constructed by placing a DNA fragment containing *FLAG(stop codon)-GFP-*T_{ADH2}*-TRP1* at the same location by gene knock-in via homologous recombination. This bicistronic construct encodes Tdh3p-FLAG, and separately GFP, but does not include an *IGG* sequence to couple the translation of the two gene partners in the operon. **a.** Western blot analysis of Tdh3p (detected with the anti-FLAG antibody). Lane 1, Strain *TDH3-fusion-GFP* (Tdh3p-FLAG-GFP detected at 64 kDa); lane 2: *TDH3::GFP* (Tdh3p-FLAG detected at 37 kDa); lane 3: protein size marker. **b.** GFP expression as observed by fluorescence microscopy. The scale bars for the images are 20 μm.

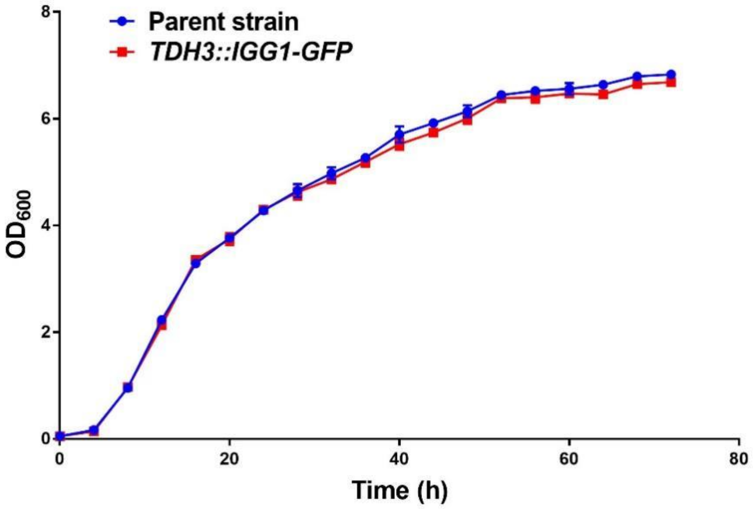

Supplementary Fig. 2. Growth of parent strain *Saccharomyces cerevisiae* CEN.PK 2-1D and the engineered strain *TDH3::IGG1-GFP*.

Data and error bars reflect the mean and the standard deviation of three independent biological replicates. Source data are provided as a Source Data file.

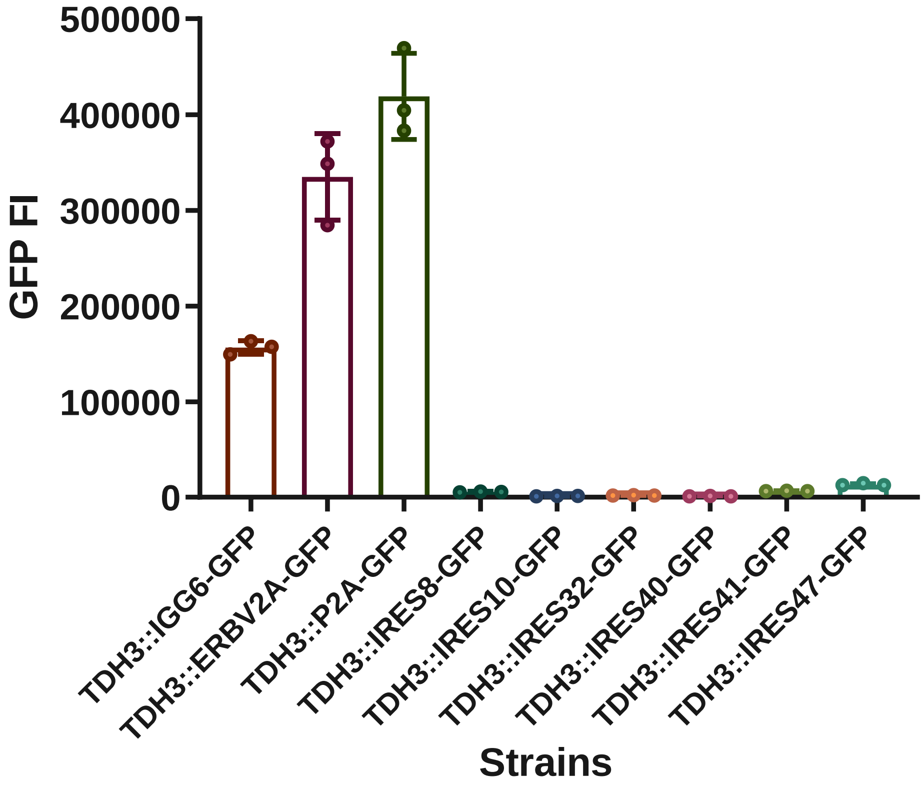

Supplementary Fig. 3. Comparison of polycistronic expression mediated by *IGG6,* 2A peptides or IRESs in *Saccharomyces cerevisiae*.

Two highly efficient 2A peptides (ERBV-1 2A and P2A) and six of the most active IRESs (IRES-8, IRES-10, IRES-32, IRES-40, IRES-41, and IRES-47) were selected from previous studies [9,10]. To form the bicistrons, DNA fragments containing *FLAG(stop codon)-IGG6-GFP-*T_{ADH2}*-TRP1*, or *FLAG(stop codon)-*$IRES_X$*-GFP-*T_{ADH2}*-TRP1* were introduced to replace of the stop codon and 45 bp of the 3' UTR of the genomic copy of the *TDH3* gene in parent strain *S. cerevisiae* CEN.PK2-1D. The DNA sequence *FLAG(no stop codon)-*$2A_X$*-GFP-*T_{ADH2}*-TRP1* encoding the 2A peptide – GFP chimera was placed to the same position of the *TDH3* gene, without a stop codon separating the Tdh3p-FLAG and GFP fusion partners. Data and error bars show the mean and the standard deviation for three independent biological replicates. Source data are provided as a Source Data file.

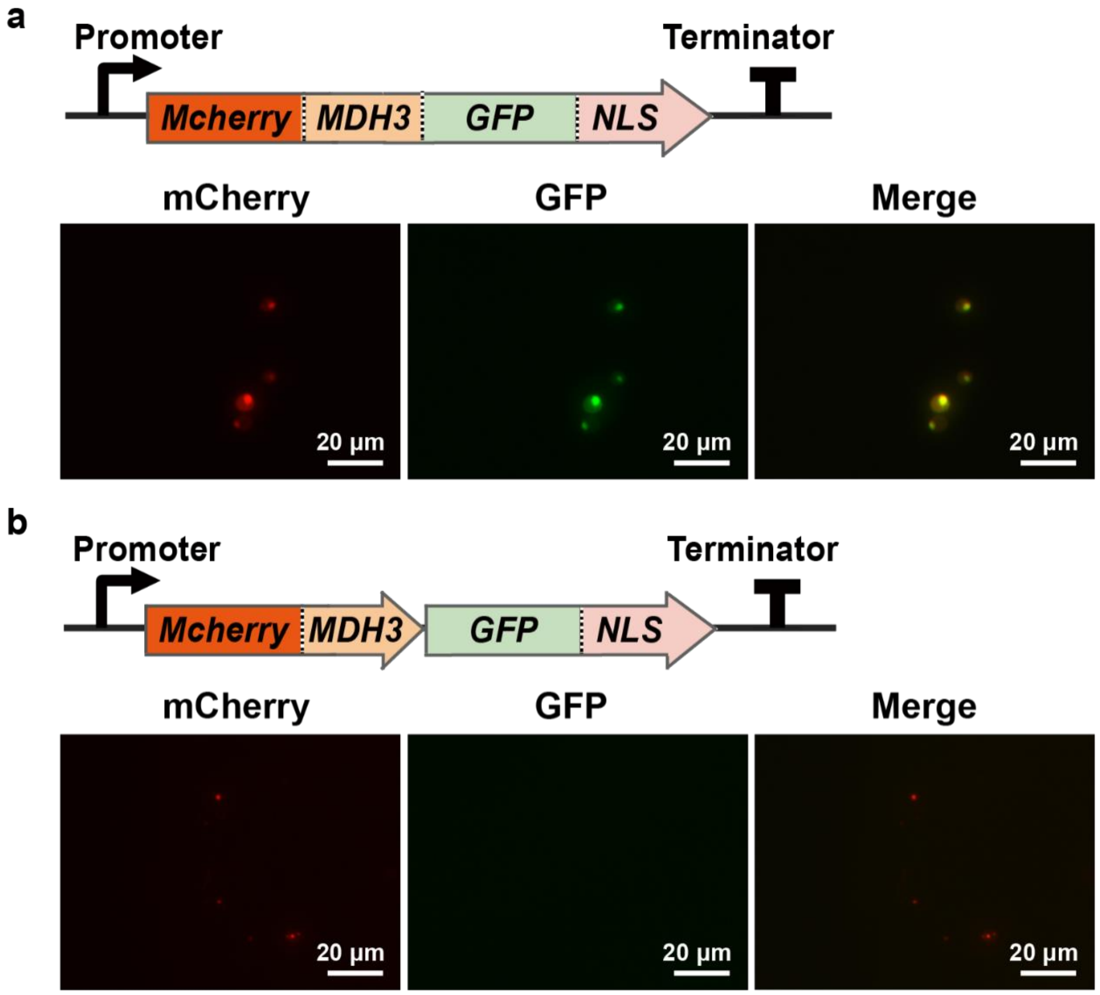

Supplementary Fig. 4. Expression of mCherry and GFP in the *mCherry-fusion-GFP* and *mCherry::GFP* transformants as observed by fluorescence microscopy.

a. The *mCherry-fusion-GFP*, without *IGG* or a stop codon between *mCherry-MDH3* and *GFP*, generated an mCherry-MDH3-GFP-NLS fusion protein, and mCherry and GFP signals were observed co-localized in the nucleus. **b.** In strains carrying the *mCherry::GFP* construct that contained a stop codon but no *IGG* between *mCherry-MDH3* and *GFP*, the mCherry signal was present in the peroxisome, but no GFP signal was observed. The scale bars for the images are 20 μm.

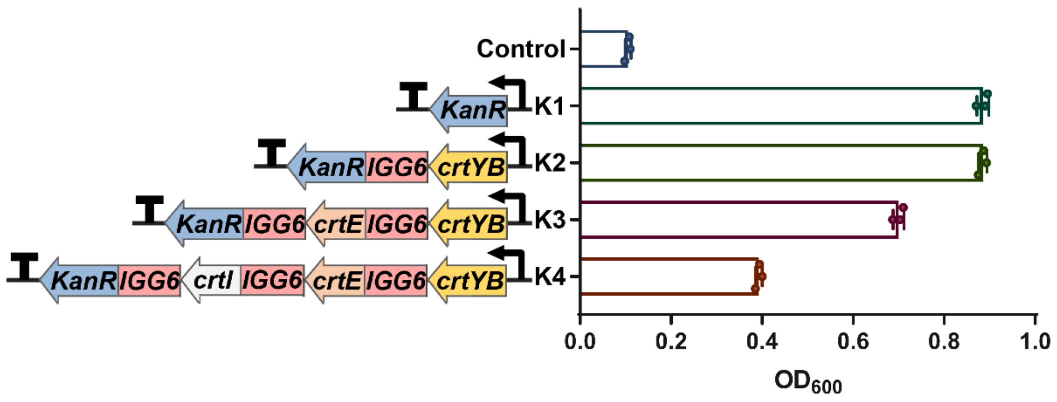

Supplementary Fig. 5. Cell growth as an indicator of the production of a functionally active antibiotic resistance determinant from a polycistronic expression cassette.

The kanamycin resistance protein (KanR from *Streptococcus pneumoniae*, UniProt ID: A0A1S6TLG9) was adopted as a reporter. Growth of *Saccharomyces cerevisiae* cells in YPD medium with Zeocin (400 µg mL^{-1}) verified the expression of *KanR*. OD_{600} values and error bars reflect the mean and the standard deviation of three independent biological replicates. The carotenoid biosynthetic genes *crtYB*; or *crtYB* and *crtE*; or *crtYB*, *crtE* and *crtI* were included to encode a first; or a first and second; or a first, second and third translation unit in polycistronic constructs K2, K3 or K4, respectively. The production of *β*-carotene and phytoene was confirmed in the engineered yeast K4 that carries the tetrascistron with the carotenoid biosynthetic genes *crtYB*, *crtE*, *crtI*, together with *KanR* (Supplementary Fig. 6). This validated the expression of the first three ORFs in addition to *KanR* [11]. Source data are provided as a Source Data file.

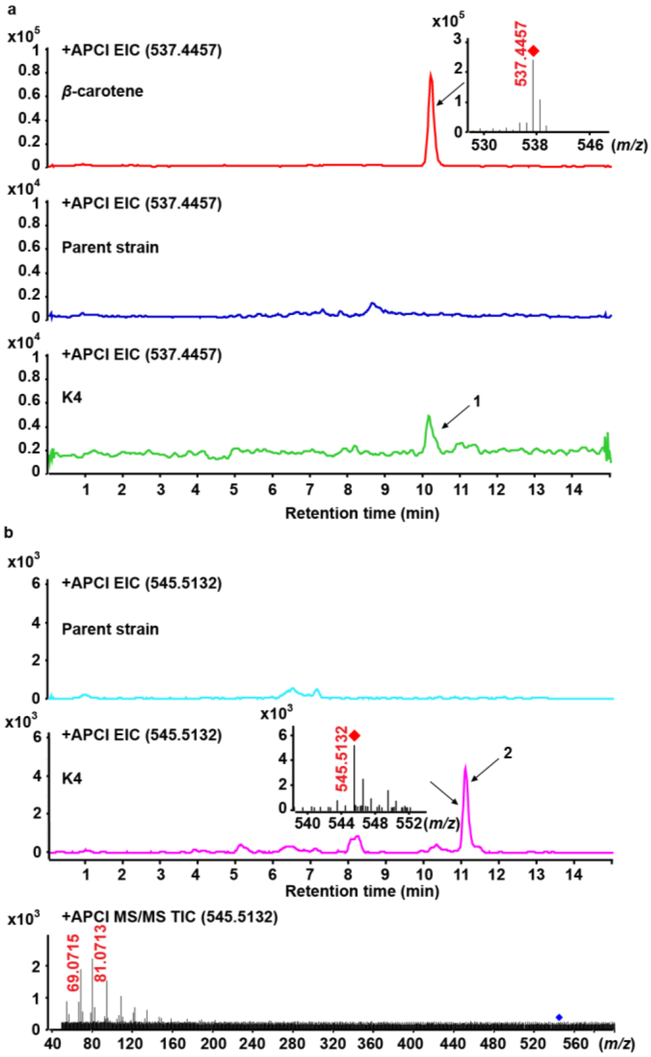

Supplementary Fig. 6. Production of *β*-carotene and phytoene in the engineered strain K4 in shake flask fermentations after 2 days of cultivation.

a. LC-HRMS profiles of a *β*-carotene authentic standard, and fermentation extracts of the parent strain and strain K4 that expresses *crtYB*, *crtE*, *crtI*, and *KanR* as a polycistron. Based on the retention time and the molecular ion, peak 1 was identified as *β*-carotene. **b.** LC-HRMS/MS profiles of phytoene in fermentation extracts of the parent strain and strain K4. The mass spectrum of peak 2 showed the molecular ion at *m/z* 545.5132 (mass error 8.4 ppm), and daughter ions in the MS/MS spectrum at *m/z* 81.0713 and 69.0715. Peak 2 was identified as phytoene by comparing these values and the retention time of the peak with the corresponding literature data [12].

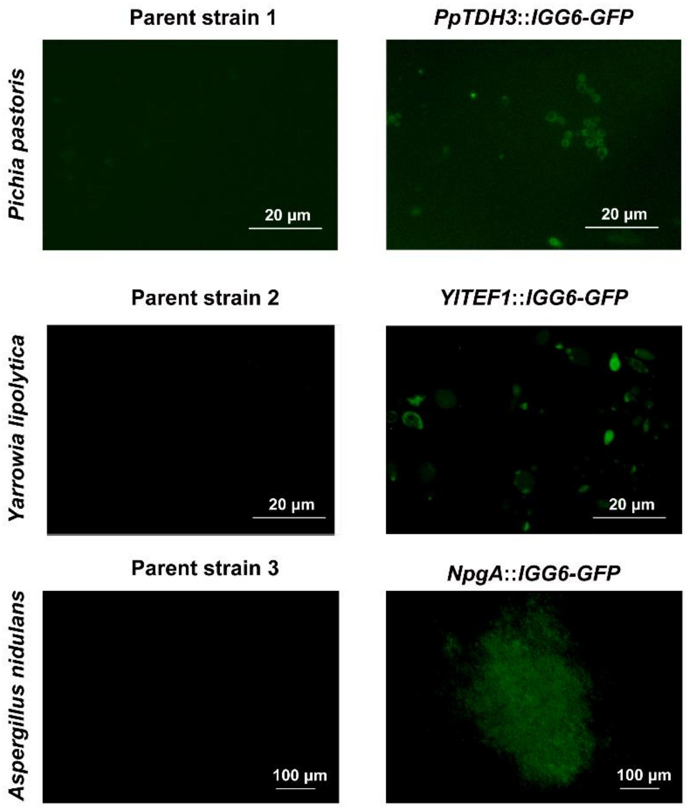

Supplementary Fig. 7. GFP production from bicistronic expression units in various fungi.

GFP fluorescence images of cultures of *Pichia pastoris* GS115, *Yarrowia lipolytica* PO1f and *Aspergillus nidulans* A1145 ΔEMΔST parent strains, and their transformants (Supplementary Data 8) carrying *IGG6*-linked bicistronic GFP cassettes, as observed by fluorescence microscopy. The scale bars for the images of *P. pastoris* and *Y. lipolytica* are 20 µm, and those for the images of *A. nidulans* are 100 µm.

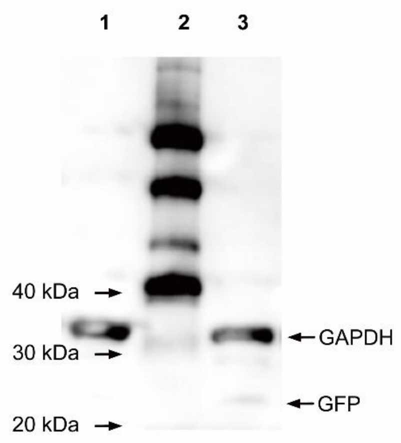

Supplementary Fig. 8. Western blot analysis of GFP expression in the transformants of *Pichia pastoris* carrying an *IGG6*-linked bicistronic GFP cassette.

The Western blot shows the expression of GFP (~27 kDa, detected by an anti-GFP rabbit polyclonal antibody [Huaxingbio, China]), and GAPDH (~36 kDa, used as a loading control and detected by an anti-GAPDH rabbit polyclonal antibody [Huaxingbio, China]). Lane 1, parent strain *P. pastoris* GS115; lane 2, protein marker; lane 3, *PpTDH3::IGG6-GFP* transformant.

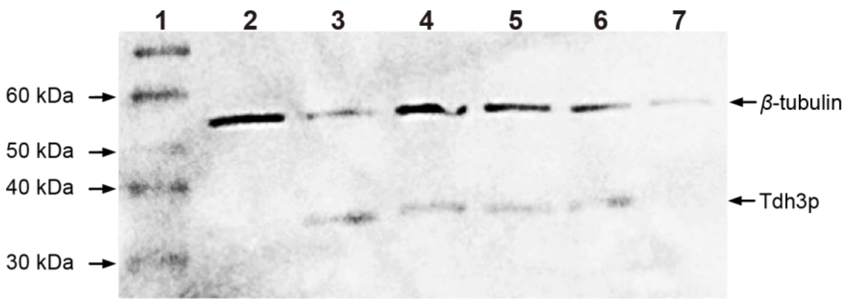

Supplementary Fig. 9. Expression of Tdh3p in the transformants presented in Figure 2.

Western blot analysis of Tdh3p. The Western blot shows the expression of Tdh3p (~37 kDa, detected by an anti-FLAG-tag rabbit polyclonal antibody [Huaxingbio, China]), and *β*-tubulin (~55 kDa) which was used as a loading control and detected by an anti-*β*-tubulin rabbit polyclonal antibody (Huaxingbio, China). Lane 1, protein marker; lane 2, parent strain *Saccharomyces cerevisiae* CEN.PK 2-1D; lane 3, *TDH3-FLAG1* transformant; lane 4, *TDH3::IGG6-GFP* transformant; lane 5, *TDH3::IGG6-TBS-GFP* transformant; lane 6, *TDH3::TBS-IGG6-GFP* transformant; lane 7, *TBS-TDH3::IGG6-GFP* transformant. TBS, translation blocking sequence.

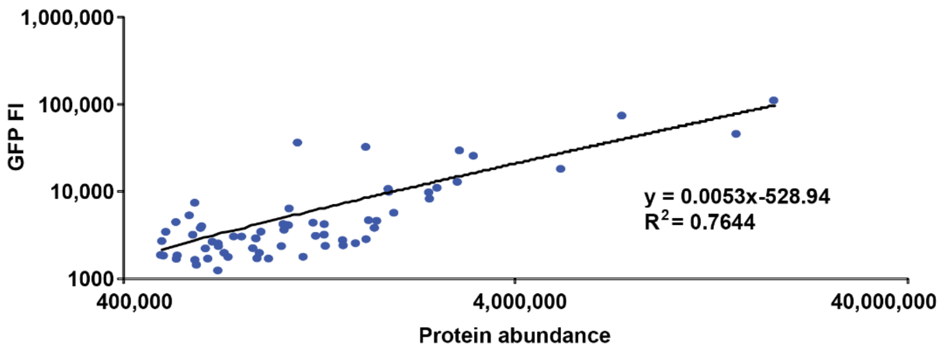

Supplementary Fig. 10. Correlation between the fluorescence intensity (FI) of the hitched GFP and the protein abundance of its endogenous driver gene.

All FIs and protein abundances are reported as the mean of three biological replicates. y=0.0053x-528.94, R^2=0.7644. Source data are provided as a Source Data file.

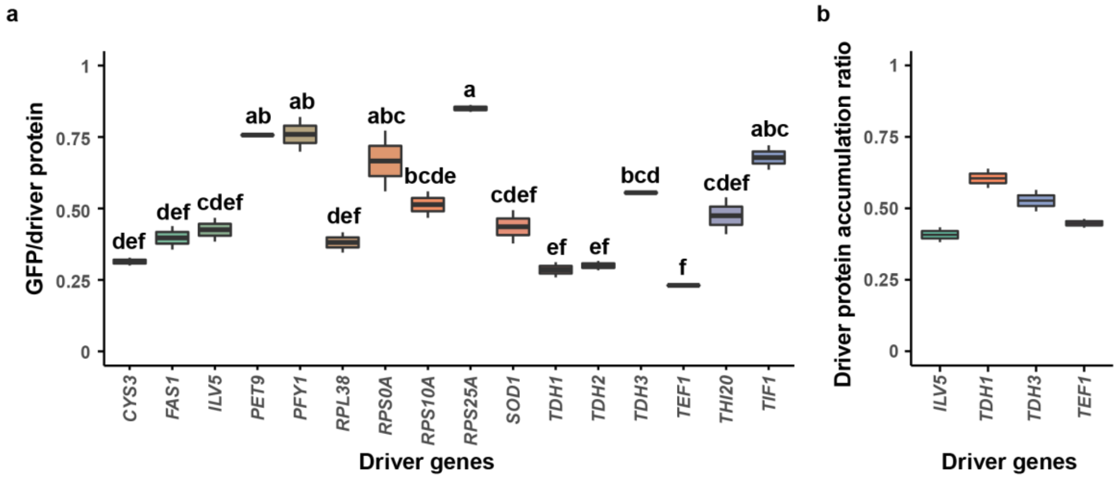

Supplementary Fig. 11. ELISA analysis of driver-FLAG and GFP.

a. The ratio of GFP accumulation and the accumulation of its driver protein in *driver-FLAG::IGG6-GFP* constructs. ELISA kits for GFP (Cloud-Clone Corp., China) or FLAG (FineTest, China) were used to detect GFP or FLAG-containing driver proteins, respectively. Box plots indicate median (middle line), 25th, 75th percentile (box) and 5th and 95th percentile (whiskers). n = 2 biologically independent replicates. Statistical analysis was performed by one-way ANOVA test with Tukey Pairwise Comparisons (95% Confidence). Means that do not share a letter are significantly different ($P < 0.05$). **b.** The ratio of the accumulation of the Driver-FLAG protein in *driver-FLAG::IGG6-GFP* transformants versus that in the corresponding *driver-FLAG* transformants. Box plots indicate median (middle line), 25th, 75th percentile (box) and 5th and 95th percentile (whiskers). n = 2 biologically independent replicates. Source data are provided as a Source Data file.

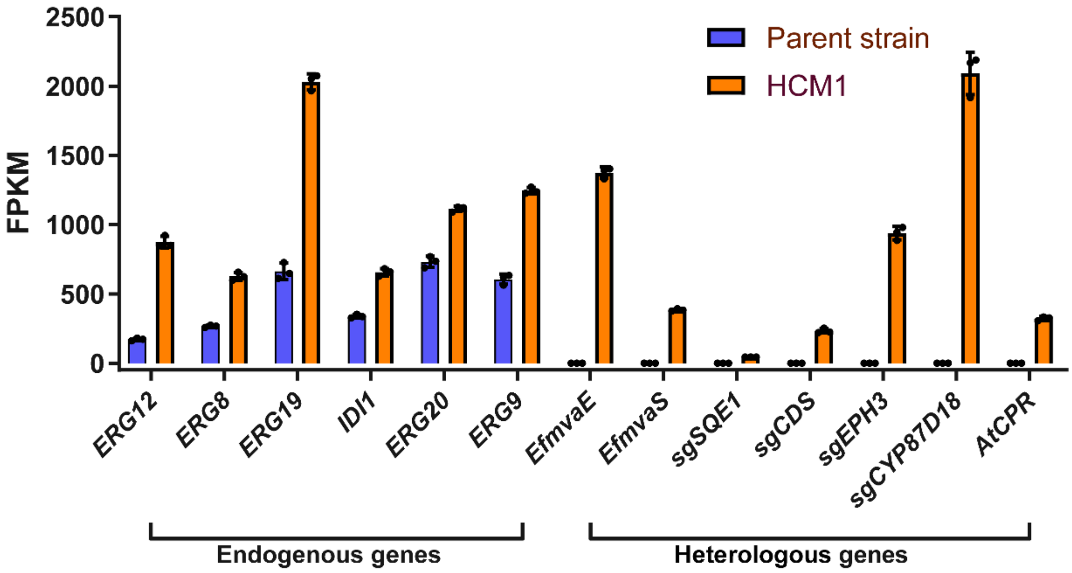

Supplementary Fig. 12. Relative expression levels of the integrated mogrol biosynthetic genes.

The engineered HCM1 strain was compared with the parental *Saccharomyces cerevisiae* CEN.PK2-1D using RNA-seq. FPKM: Fragments per kilobase of exon per million mapped fragments. Data and error bars show the mean and standard deviation of three independent biological replicates. Source data are provided as a Source Data file.

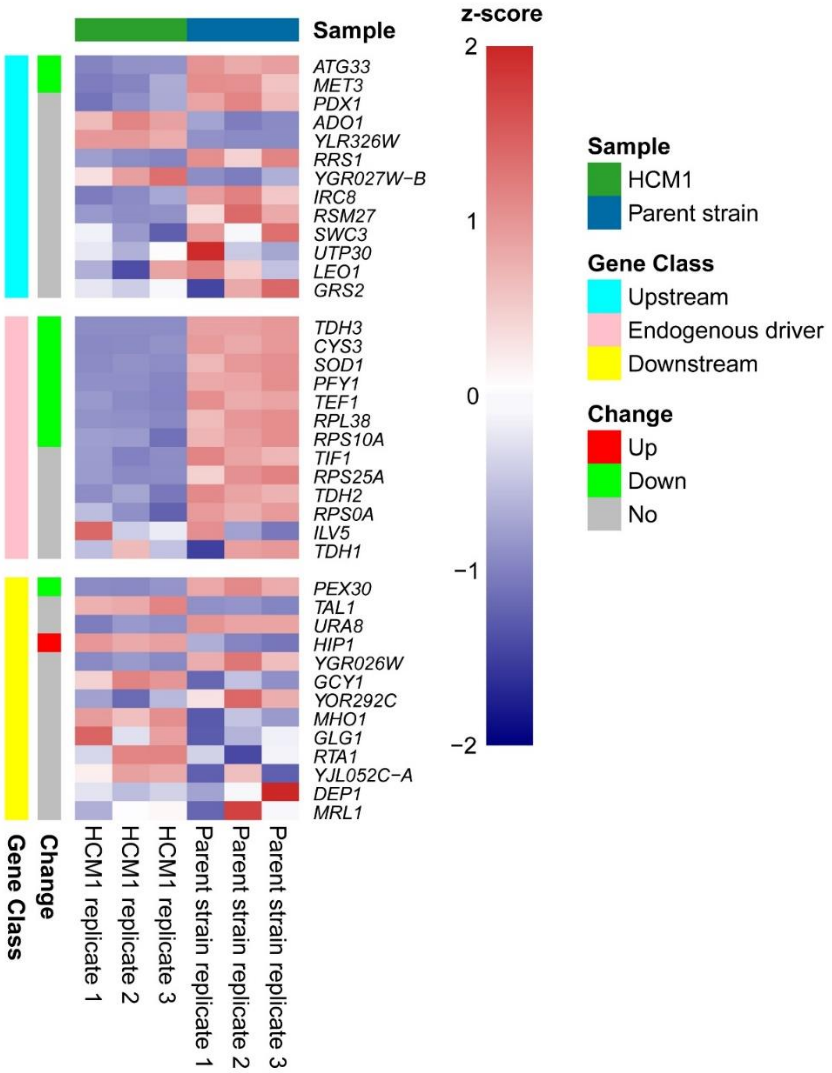

Supplementary Fig. 13. Transcriptome analysis of the HACKing-engineered strain HCM1.

The heatmap shows the gene expression (z-scaled FPKM) of selected genes. Genes that are differentially expressed (p adj <0.05, |log2_ratio|>1) between the mogrol-producing *Saccharomyces cerevisiae* HCM1 and the parental strain CEN.PK2-1D are indicated in the *Change* bar to the left of the heatmap: *Red* indicates relative upregulation of gene expression; *gray* indicates no significant change; and *green* shows relative downregulation of gene expression. *Gene Class*: The 13 endogenous driver genes are labeled with a *pink bar*, and their upstream and downstream gene neighbors on the yeast chromosome are labeled with *cyan* and *yellow bars*, respectively.

Supplementary references

1 Reider Apel, A. et al. A Cas9-based toolkit to program gene expression in *Saccharomyces cerevisiae*. *Nucleic Acids Res.* **45**, 496-508 (2017).

2 Manivasakam, P., Weber, S. C., McElver, J. & Schiestl, R. H. Micro-homology mediated PCR targeting in *Saccharomyces cerevisiae*. *Nucleic Acids Res.* **23**, 2799-2800 (1995).

3 Zhang, Y. et al. A gRNA-tRNA array for CRISPR-Cas9 based rapid multiplexed genome editing in *Saccharomyces cerevisiae*. *Nat. Commun.* **10**, 1053 (2019).

4 Jinek, M. et al. A programmable dual-RNA-guided DNA endonuclease in adaptive bacterial immunity. *Science* **337**, 816-821 (2012).

5 Xie, L. et al. Methylglucosylation of aromatic amino and phenolic moieties of drug-like biosynthons by combinatorial biosynthesis. *Proc. Natl. Acad. Sci. USA* **115**, E4980-E4989 (2018).

6 Yue, Q. et al. Functional operons in secondary metabolic gene clusters in *Glarea lozoyensis* (Fungi, Ascomycota, Leotiomycetes). *mBio* **6**, e00703-15 (2015).

7 Ivanov, P. A. et al. A tobamovirus genome that contains an internal ribosome entry site functional in vitro. *Virology* **232**, 32-43 (1997).

8 Yin, W. B. et al. A nonribosomal peptide synthetase-derived iron(III) complex from the pathogenic fungus *Aspergillus fumigatus*. *J. Am. Chem. Soc.* **135**, 2064-2067 (2013).

9 Souza-Moreira, T. M. et al. Screening of 2A peptides for polycistronic gene expression in yeast. *FEMS Yeast Res.* **18**, foy036 (2018).

10 Zhou, W., Edelman, G. M. & Mauro, V. P. Isolation and identification of short nucleotide sequences that affect translation initiation in *Saccharomyces cerevisiae*. *Proc. Natl. Acad. Sci. USA* **100**, 4457-4462 (2003).

11 Xie, W., Lv, X., Ye, L., Zhou, P. & Yu, H. Construction of lycopene-overproducing *Saccharomyces cerevisiae* by combining directed evolution and metabolic engineering. *Metab. Eng.* **30**, 69-78 (2015).

12 Rivera, S., Vilaro, F. & Canela, R. Determination of carotenoids by liquid chromatography/mass spectrometry: effect of several dopants. *Anal. Bioanal. Chem.* **400**, 1339-1346 (2011).
