# Supplementary material for: A polycistronic system for multiplexed and precalibrated expression of multigene pathways in fungi
Source: Nat Commun. 2023 Jul 17;14:4267. doi: 10.1038/s41467-023-40027-0 (PMC10352335; doi:10.1038/s41467-023-40027-0)
Supplement: Supplementary file 3 — Description of Additional Supplementary Files [file 41467_2023_40027_MOESM3_ESM.pdf]

Description of Additional Supplementary Files

File Name: Supplementary Data 1
Description: Intensity-based absolute quantification of CEN.PK2-1D proteins by proteomic analysis.

File Name: Supplementary Data 2
Description: : Available target driver genes for direct genomic integration into *S. cerevisiae* using the CRISPR/Cas9 system.

File Name: Supplementary Data 3
Description: List of gRNA used in this study.

File Name: Supplementary Data 4
Description: Endogenous driver genes in *S. cerevisiae* shown in Fig 3c and their guide sequences.

File Name: Supplementary Data 5
Description: Production of squalene and mogrol in the engineered yeasts.

File Name: Supplementary Data 6
Description: Differentially expressed genes in HCM1 relative to the parent strain CEN.PK2-1D.

File Name: Supplementary Data 7
Description: List of plasmids used in this study.

File Name: Supplementary Data 8
Description: List of strains used in this study.

File Name: Supplementary Data 9
Description: List of primers used in this study.

File Name: Supplementary Data 10
Description: DNA sequences of the enzymes used in the squalene and mogrol pathways engineered in this work.
